# Research on the Design and Alignment Method of the Optic-Mechanical System of an Ultra-Compact Fully Freeform Space Camera

**DOI:** 10.3390/s23239399

**Published:** 2023-11-25

**Authors:** Yunfeng Li, Zongxuan Li, Tiancong Wang, Shuping Tao, Defu Zhang, Shuhui Ren, Bin Ma, Changhao Zhang

**Affiliations:** 1 Changchun Institute of Optics, Fine Mechanics and Physics, Chinese Academy of Sciences, Changchun 130033, China; liyunfeng18@mails.ucas.ac.cn (Y.L.); wtcshuaige@sina.com (T.W.); taoshuping-163@163.com (S.T.); zdf713@163.com (D.Z.); ryougiit@gmail.com (S.R.); binma2020@outlook.com (B.M.); zch9905@163.com (C.Z.); 2 University of Chinese Academy of Sciences, Beijing 100049, China; 3 Key Laboratory of Space-Based Dynamic & Rapid Optical Imaging Technology, Chinese Academy of Sciences, Changchun 130033, China

**Keywords:** space optical camera, freeform optical systems, integrated optomechanical analysis, freeform optical systems

## Abstract

As space resources become increasingly constrained, the major space-faring nations are establishing large space target monitoring systems. There is a demand for both the number and the detection capability of space-based optical monitoring equipment. The detection range (i.e., field of view) and parasitic capability (lightweight and small size) of a single optical payload will largely reduce the scale and cost of the monitoring system. Therefore, in this paper, the optic-mechanical system of an ultra-lightweight and ultra-compact space camera and the optical alignment method are investigated around a fully freeform off-axis triple-reversal large field of view (FOV) optical system. The optic-mechanical system optimisation design is completed by adopting the optic-mechanical integration analysis method, and the weight of the whole camera is less than 10 kg. In addition, to address the mounting problems caused by the special characteristics of the freeform surface optical system, a dual CGH coreference alignment method is innovatively proposed. The feasibility of the method is verified by the mounting and testing test, and the test results show that the system wavefront difference is better than 1/10 λ. The imaging test of the space camera and the magnitude test results meet the design requirements of the optical system. The optic-mechanical system design method and alignment method proposed in this paper are instructive for the design and engineering of large field of view full freeform optical loads.

## 1. Introduction

Technological advances have led to the expansion of human exploration from the surface of the Earth to the vastness of space, which has become an important strategic resource [1]. The major spacefaring nations are sparing no effort in carrying out various space activities in an endeavour to occupy the high ground in space technology. As the mainstay of space activities, various artificial satellites and manned spacecraft, while carrying out space missions, are also threatened by various space targets, including space debris [2,3,4]. In order to safeguard space interests and space security, it is necessary to obtain the current operational status of space targets and to control the future activity trends of space targets, i.e., space situational awareness. Currently, the most effective way of sensing space dynamics is through a network of space-based observation equipment to continuously track and record space targets [5]. The United States, Russia, and a number of countries in Europe are building their own space surveillance networks [6]. The United States was the earliest country in the world to carry out research on space target observation, and it has mainly built the Space-Based Infrared System (SBIRS) [7], the Midcourse Space Experiment Project (MSX) [8], and the Space-Based Space Surveillance (SBSS) [9,10,11], and so on. The survey counted the field of view parameters of space target surveillance cameras in various countries, as shown in Table 1. From Table 1, it can be found that the field of view angles are mostly below 3° × 3°. The observation capability of a single space-based optical payload will have a direct impact on the size and performance of the space-based surveillance network, i.e., the farther and wider a single space-based optical payload can see, the more it will greatly reduce the size and cost of the system network. Therefore, the study of ultra-compact, large field of view optical payloads with excellent detection performance is of great significance for space situational awareness missions.

Therefore, this article investigates the challenges in the design and engineering implementation of an ultra-compact, fully freeform optical load with a large field of view, using a space camera as the object of study. The optic-mechanical system is optimally designed based on the optic-mechanical integration analysis method, which solves the contradiction between lightweight and dimensional instability of the optic-mechanical system. Aiming at the problem of difficulty in mounting and adjusting the special-shaped optical system, the system integration scheme of double CGH common reference is proposed, and the mounting and adjusting work of the shaped optical system is successfully completed. The final indicators have met the design requirements of the optical system. This article focuses on the full freeform special configuration optical system, which has new breakthroughs in the analysis of optic-mechanical system integration and system mounting. This paper provides a new technical solution for the development of an ultra-compact space target detection optical payload with a large field of view. At the same time, the application of this optical payload is expected to be of great significance for the miniaturisation of the network scale of the space surveillance system.

## 2. Introduction to Full Freeform Optical Systems

Firstly, the metric requirements of the optical payload are defined according to the exploration mission. The optical payload is planned to probe downward in a 100,000 km orbit to acquire information about space targets larger than 0.4 m in a 30,000 km orbit. The optical payload should possess the capability to acquire information from space targets in the ultraviolet (350–400 nm), visible light (400–900 nm), and near-infrared (800–1000 nm) spectral bands while also having a large field of view detection capability of 4° × 4°. The limiting magnitude index of the camera can be calculated based on the relationship between the detection target area, the detection distance, and the equivalent magnitude [12]. The size of the detection target is 0.4 m, its area is 0.16 m^2^, and the calculated limiting magnitude of the optical system should be not less than 14th magnitude, which can meet the detection requirements.

**Figure 1:**
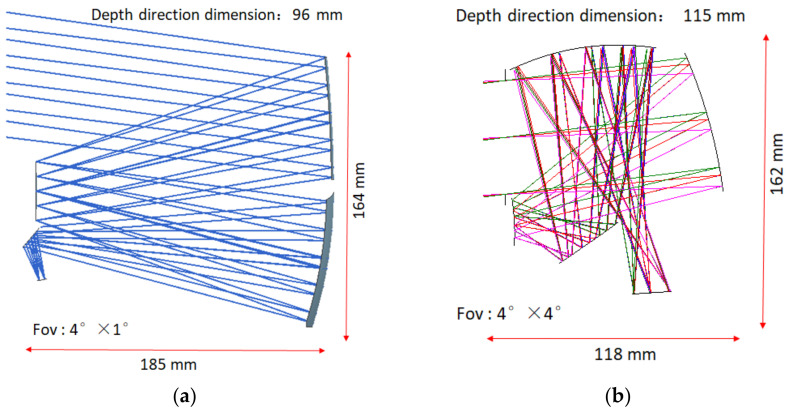
Schematic diagram of an off-axis triplex optical system: (**a**) conventional off-axis three-reflector system structure form; (**b**) tilt-biased fully freeform off-axis triple-reflector system structural form designed in this paper.

In order to achieve the mission requirements of a large field of view and ultra-compactness for optical loads, the large field of view metrics can generally be achieved with coaxial transmission systems and off-axis reflection systems. Transmission systems have the disadvantages of chromatic aberration, poor thermal stability, narrow spectral range, and difficulty in suppressing stray light compared to reflection systems [13,14]. The objective of this project is to obtain more target information, therefore requiring the camera to have a wide spectral response range. The priority is given to the off-axis system. The off-axis system can be further classified into coaxial and off-axis systems, with the latter being more suitable for achieving a large field of view. Therefore, the off-axis system is more suitable for the implementation of this project. In the field of space cameras, off-axis two-mirror and off-axis three-mirror systems are widely used. The off-axis three-mirror system has several advantages over the off-axis two-mirror system: (1) The three-mirror system has more reflection surfaces, making it easier to achieve a compact design in terms of the overall dimensions. (2) The three-mirror system has an additional reflection mirror, providing greater flexibility for the design and optimisation of the optical system. Therefore, the off-axis three-mirror system is selected as the initial configuration of the optical system for this project. The conventional off-axis triple-reflector system has a large outer envelope size. According to the requirements of the mission index, we used the traditional off-axis triple-reflector structural form for the optical system design, and the design results are shown in Figure 1a. The design results show that this solution not only has a large outer envelope size but also cannot achieve the 4° × 4° field of view requirement. Therefore, in this paper, a tilt-biased fully freeform off-axis triple-reflector system is proposed, as shown in Figure 1b. The system uses freeform surfaces for all optical mirrors. Compared to the secondary surfaces used in previous reflection systems, the use of all freeform surfaces not only increases the optimisation space of the optical system but also solves the system distortion and aberration problems brought on by the large field of view. The design parameters of the final optical system are shown in Table 2.

Although the detection requirements such as a large field of view and ultra-compactness of optical payloads have been met in the design of the optical system, the design of the optic-mechanical system, the optical inspection, and the system mounting will bring about new challenges. In terms of optic-mechanical system design, the first step is to solve the contradiction between the lightweight design of the optic-mechanical system and the stability support. The system requires that the weight of the whole machine is less than 10 kg, and at the same time, it should ensure that the shape accuracy of the reflector assembly is better than 1/40 λ under the working conditions of ±5 °C and gravity. In terms of optical inspection, the inspection of freeform optical mirrors cannot be inherited from the traditional spherical or quadratic surface inspection methods, so a new inspection scheme needs to be proposed. In terms of system tuning, the special configuration of the optical system cannot be aligned by traditional tuning, and it is necessary to further solve the tuning problem of the special configuration of the optical system so that the wave aberration of the final system will be better than 1/10 λ and the camera can detect the stars at 14th magnitude. The following is a detailed discussion centred on both of these aspects.

## 3. Optic-Mechanical System Design and Integration Analysis

Space cameras are subjected to complex external loads during both the launch segment and the in-orbit operation phase, which include gravitational release into orbit and temperature changes during in-orbit operation. These external loads lead to destabilisation of the optical mirrors and degradation of the imaging performance of the optical system [15,16,17]. Therefore, the force thermal stability of the optic-mechanical system will be one of the core components of the optic-mechanical system design. Another design optimisation is centred on how to achieve ultra-lightweight and ultra-compact space optical loads. In terms of space system applications, an ultra-lightweight and ultra-compact optical payload facilitates the layout of the entire satellite and can also be carried as a parasitic payload in multiple satellite systems to achieve rapid networking in orbit. At the whole satellite level, ultra-lightweight and ultra-compact payloads have a positive impact on the cost of the launch, on-orbit fuel consumption, and on-orbit lifetime.

### 3.1. Selection of Materials

For reflective optical systems, commonly used optical mirror materials for aerospace applications include silicon carbide, aluminium metal, microcrystalline glass, monocrystalline silicon, quartz glass, beryllium metal, and so on. The selection of mirror materials will take into account the density, thermal expansion coefficient, mechanical processability, optical processability, thermal conductivity, modulus of elasticity, and other material properties according to the application requirements. The properties of the materials commonly used in space optical mirrors are shown in Table 3.

In Table 3, it can be seen that all the properties of silicon carbide material are excellent but the mechanical processability is poor, resulting in a long cycle time for the preparation and milling of the mirror blank. Therefore, it is difficult to achieve quick integration and application of this payload. The thermal stability of microcrystalline glass is relatively outstanding, but its low modulus of elasticity and poor mechanical stability are not suitable for an optic-mechanical system ultra-lightweight design. Beryllium aluminium alloys have good force thermal stability, but the material itself is toxic, posing some challenges to the development process. For the aluminium alloy material, the force thermal performance is moderate, with good mechanical and optical machinability. These features mean that the ultra-lightweight design of the optical machine structure can be realised, and at the same time, using the single-point turning process, the face shape precision of the mirror can quickly reach 1/20 λ, which can save a lot of time for the subsequent fine polishing. Therefore, the preparation and processing costs of aluminium mirrors will be the lowest, and it will be very practical for quick responses in war. After comprehensive consideration, aluminium alloy 6061 was finally selected as the reflector material.

The choice of materials for the camera frame also revolves around the issue of mechanical and thermal stability. Preference was given to materials with high specific stiffness for mechanical stability and low coefficients of thermal expansion and high thermal conductivity for thermal stability. Aluminium alloys, titanium alloys, and carbon fibres are commonly used for camera frames. Titanium alloys and carbon fibres have high specific stiffness but poor thermal conductivity. This section of material is mainly applied to optical loads that are not sensitive to thermal loads. This system involves multiple spectral regions, including ultraviolet, visible light, and near-infrared. The uneven temperature caused by the poor thermal conductivity of the materials will greatly reduce the imaging performance of the optical system. The thermal conductivity and mechanical properties of aluminium alloy materials are acceptable compared to titanium alloy and carbon fibre as the camera frame structure for this load. However, the stiffness-to-density ratio of aluminium alloy is average, and the stiffness determines the degree of lightweight of the camera frame. Therefore, we will introduce a material called aluminium-based high-body component composites that have higher stiffness, comparable mechanical machinability, and thermal stability.

It is an aluminium matrix composite material reinforced with silicon carbide particles, which has a very rich application scenario in aerospace and aviation [18]. Its main advantages are as follows: (1) In mechanical properties, it has double the specific stiffness than aluminium alloy and titanium alloy. (2) In thermal properties, it has a coefficient of thermal expansion close to beryllium material and a dimensional stability superior to that of beryllium material. Therefore, the optic-mechanical system’s force and thermal stability can be ensured by choosing the high-body component composite material as the camera frame material.

### 3.2. Optimised Design of the Optical Components

The structural layout of the optic-mechanical system is carried out first. The system structure layout is around the structure of the optical system form, and it should not only consider the weight of the camera, the size of the outer envelope, mechanical properties and other indicators but also take into account the material structure of the processing technology and the feasibility of the system tuning programme. Figure 1b shows that this system is an off-axis triple-reflector optical system, and most of the traditional off-axis triple-reflector systems use frame and truss structures. The frame-type structure is generally formed by splitting and splicing or one-piece moulding to form a box structure, and then, each mirror assembly and focal plane assembly are mounted on the frame structure, which, in turn, forms the whole optic-mechanical system. The truss structure form is assembled with rod structures and adapters for the mounting of individual optical machine components. Truss structures have a great advantage over frame structures in terms of light weight. The most famous telescope, the Hubble Telescope, uses a truss structure to support its components [19].

**Figure 2:**
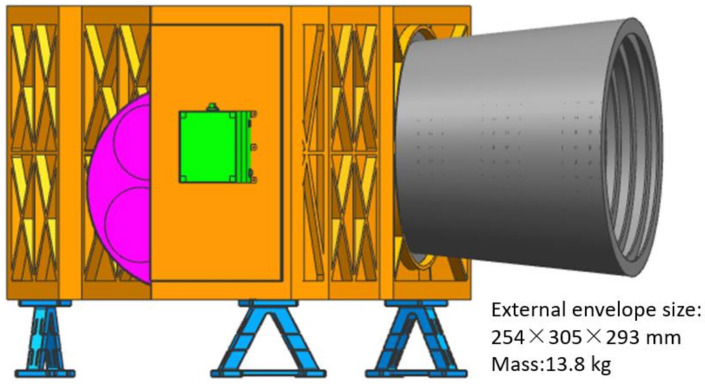
Design results of the framed structure.

However, the truss structure form is mainly applicable to optical systems with a relatively dispersed layout and a large distance between the mirrors, which is not suitable for the application of the ultra-compact optical system proposed in this study. Therefore, we conducted preliminary design work on the opto-mechanical structure around the optical system of this project using a frame structure, and the design result is shown in Figure 2. Not only does this form of construction fail to meet the 10 kg weight requirement but it also has a serious weakness. The structure is box-like, which will obstruct the detection light path during the optical system alignment process and is not suitable for the application of this system. Therefore, it is necessary to propose a new camera structure form.

**Figure 3:**
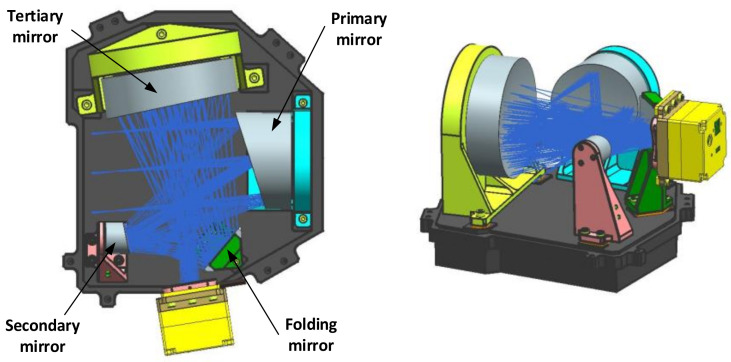
Structural layout of the optic-mechanical system.

In order to meet the needs of ultra-compactness and alignment needs, a common substrate layout structure is proposed, as shown in Figure 3. All the mirrors and focal plane components are mounted on a single substrate, which, in turn, realises the co-substrate mounting of each component. Compared to the scheme in Figure 2, this structural configuration adopts a fully open design, which greatly facilitates the integration and assembly of opto-mechanical systems. Furthermore, it possesses a reduced envelope size, thus enabling a more compact layout for opto-mechanical systems.

**Figure 4:**
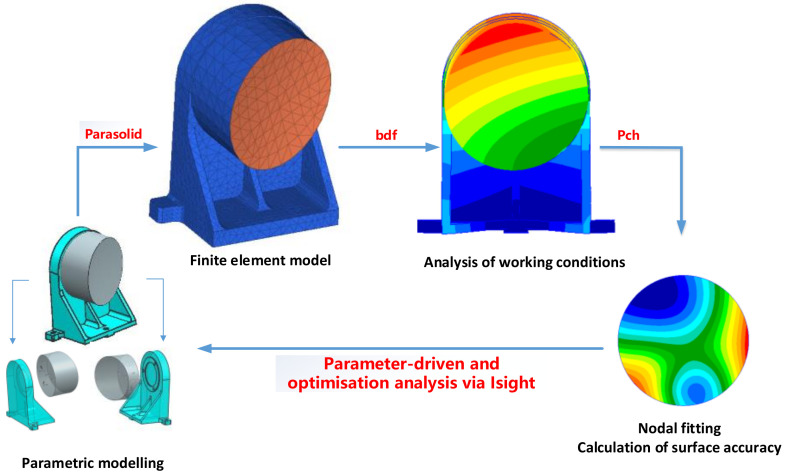
Analysis and optimisation process for the opto-mechanical integration of reflector assemblies.

After completing the layout of the optic-mechanical system, the design of the mirror assembly needs to be carried out. The design of the mirror assembly is a key part of the optic-mechanical system, because the precision of the mirror’s surface shape directly affects the imaging quality of the camera system. Due to the difference between the space environment and the ground environment, the surface shape accuracy of the mirror will be affected by gravity and temperature changes. In order to meet the needs of space applications, the system requires that the surface shape accuracy of the space mirror should change within 13 nm under three directions of gravity and a 5 °C temperature rise. At the same time, the optic-mechanical system needs to be lightweight. Conventional optic-mechanical system design usually adopts an empirical design method; however, this method cannot find the balance between the optimal surface shape accuracy of the mirror and the lightweight of the optic-mechanical system, which leads to excessive redundancy of the design and waste of resources. In order to solve this problem, we adopt the design method of optomechanical integration optimisation. This approach closely integrates the mechanical design results with the optical performance index of the mirrors to achieve a closed-loop optimisation of the mirror assembly design. The specific design optimisation iteration process of the primary mirror assembly is shown in Figure 4, and the design process of other reflector assemblies in this system also adopts this optimisation method.

**Figure 5:**
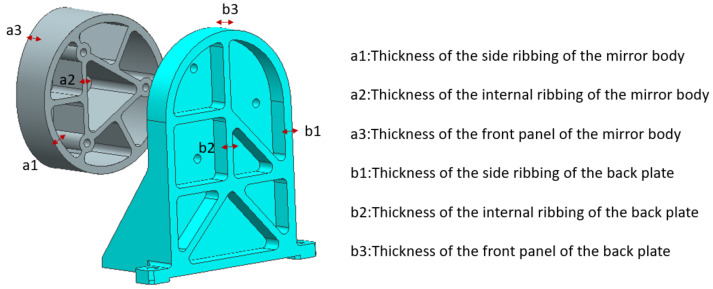
Initial optimisation model and optimisable parameters for the primary mirror assembly.

Firstly, according to the design requirements of the optical system, we designed the initial structural model of each mirror assembly. Then, we analysed the mirror nodes of these mirrors under different gravity and temperature operating conditions using the finite element method and obtained their response results. Next, we extracted the nodal displacements of the mirror surface of each mirror and fitted them using Zernike polynomials. Through this step, we obtained the variation of the mirror surface shape. Next, we took the optimised design of the main mirror as an example and introduced the optimisation process of the structural parameters of the main mirror in detail. First of all, according to the optic-mechanical system structure, the layout results confirmed the main mirror installation relationship and design space. To further design the initial configuration of the main mirror, the design results are shown in Figure 5. Then, we clarified the optimisation parameters of the mirror assembly and combined them with the processing technology to clarify the parameter range, and the corresponding design variables are shown in Figure 5. In this paper, the mirror face shape accuracy not being higher than 1/50 λ and the dynamic fundamental frequency not being lower than 120 Hz are taken as the constraints, and the minimum mass of the mirror assembly is taken as the optimisation objective in order to achieve the lightweight design of the mirror assembly under the premise that the static and dynamic stiffness meets the requirements. The design variables and optimisation results are shown in Table 4.

In the end, after the optimised design, we obtained the results of the four mirrors under different working conditions to meet the system design requirements. The specific results are shown in Table 5. Through the above method, we achieved the optimal design of the mirrors and, at the same time, ensured the applicability and reasonableness of the design results.

### 3.3. Optical-Mechanical-Thermal Integration Analysis of Optic-Mechanical Systems

**Figure 6:**
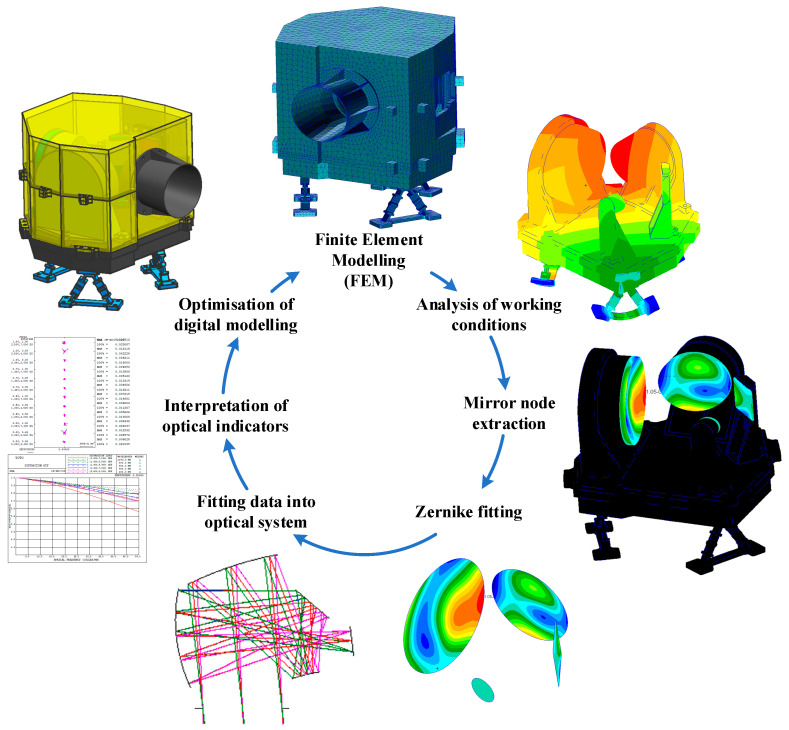
The process of optic-mechanical system optical-mechanical-thermal analyses.

After the optimal design at the optical component level is completed, it is also necessary to perform a STOP analysis on the whole optic-mechanical system. A STOP analysis is an integrated analysis method that combines structural, thermal, and optical disciplines that are capable of deterministically evaluating the optical performance of an optic-mechanical system [20,21]. Through STOP analysis, we can realise the full-link closed loop of a design, simulation, and systematic evaluation of an optic-mechanical system. Using this method, we can not only verify whether the design results meet the requirements but also provide strong support for the optic-mechanical system’s optimal design. The flow of the optical machine thermal integration analysis method is shown in Figure 6.

Firstly, finite element preprocessing is carried out on the designed camera structure, and the simulation working conditions are set according to the working environment of the camera during ground integration and in-orbit operation. Then, the finite element model of the optic-mechanical system is subjected to force and thermal analyses. By extracting the results of the displacement response of the nodes of the four mirrors in the optical system under each working condition and fitting the Zernike to the node displacements of each mirror, the relationship between the structural response and the optical parameters is established. Then, the Zernike response coefficients of each optical mirror surface are imported into the optical system, and the performance characterisation indexes of the optical system under each working condition, such as the wavefront aberration and MTF, are obtained by using the ray tracing method. Finally, the design and optimisation of the optic-mechanical system are carried out by using the optical-mechanical integration analysis method. The above is the process of analysing the data link for completing one iteration. After the optimisation of the reflector mirror assembly, the structural parameters of the mirror have been solidified. Therefore, the variables optimised for this part of the machine are the structural dimensions of the main backplate and the camera legs. The constraints of the optimisation are the thermal loads and the system wavefront difference under gravity being better than 1/10 λ, and the optimisation objective is to minimise the weight of the whole machine. Through several iterations, the finally obtained optic-mechanical system is verified by simulation, and the results are shown in Table 6. Under various working conditions, the integrated indexes of the optical system can meet the application requirements.

### 3.4. Summary

In this section, the optical-mechanical integration analysis method is primarily employed to facilitate the design of opto-mechanical systems and conduct comprehensive simulations of the opto-mechanical-thermal full chain. This approach offers considerable assistance in achieving a closed-loop design process during the early stages, accurately predicting the relationship between the optical parameters and mechanical design variables and evaluating the impact of different operational scenarios on the system. Not only does it significantly reduce the research and development time but it also ensures a rational safety margin, thereby avoiding unnecessary material and launch cost wastage.

## 4. System Integration and Testing

In the development process of a space camera, optical system mounting is an indispensable link, which has a crucial impact on the performance of the optical system. In this paper, for the special characteristics of the fully freeform off-axis spherical heterostructure optical system, a mounting method of primary and secondary mirrors with double CGH mutual tracing and common reference is proposed. The method mainly focuses on the detection principle and detection scheme formulation, and its feasibility is verified through specific experimental validation. Ultimately, the validation of the systematic wavefront aberration detection results demonstrate the applicability of the detection method in optical system mounting. This research result provides a new idea for the spherical heterostructure ultra-compact off-axis triple-reflector system integration.

### 4.1. Principle of CGH Detection

Optical inspection techniques play an important role in the processing of optical mirrors and in the assembly phase of the system. For optical systems consisting of simple quadratic surfaces, the most common inspection method is zero interferometry using a lens compensator. However, in this system, because it contains three mirrors, all of which are freeform off-axis mirrors, the traditional inspection method cannot meet its needs. Therefore, combining the processing and inspection needs of the freeform off-axis mirrors, we adopt the computational hologram method to realise the surface shape inspection of the freeform mirrors. The computer generated hologram (CGH) method is a suitable compensator for off-axis aspheric surface measurements [22,23,24].

The main working principle of computational hologram CGH is a process of wavefront recording and wavefront reproduction. The principles of wavefront recording and wavefront reproduction are described in detail in the following sections.

#### 4.1.1. Wavefront Record

Firstly, with the help of computer technology, we use a mathematical model to build a simulation of the object and detection light of an ideal optical mirror. Usually, we choose a fixed wavelength provided by a commercial laser interferometer as the light source for the detection light. Next, we apply mathematical calculations to derive and simulate the interference results of the object and detection light at the computational holographic grating (CGH) plane. Through these calculations, we are able to obtain a numerical description of the interference effects of the object light and the detected light at the CGH plane. This process provides the ability to measure and analyse the surface topography of optical mirrors in a non-contact manner.

Let the object light (containing the wavefront information of the mirror in the optical system design result) and the detection light be, respectively,

(1) $${\tilde{E}}_{o}\left(x,y\right)=a_{o}\left(x,y\right)e^{i\varphi_{o}\left(x,y\right)}$$

(2) $${\tilde{E}}_{r}\left(x,y\right)=a_{r}\left(x,y\right)e^{i\varphi_{r}\left(x,y\right)}$$

where $a_{o}\left(x,y\right)$ and $a_{r}\left(x,y\right)$ are the amplitude information of the object light and the reference light. $\varphi_{o}\left(x,y\right)$ and $\varphi_{r}\left(x,y\right)$ are the phase information of the two light beams. The light intensity distribution after the interference between the object light wave and the reference light wave is

(3) $$I\left(x,y\right)={\left|{\tilde{E}}_{o}\left(x,y\right)+{\tilde{E}}_{r}\left(x,y\right)\right|}^{2}=a_{r}{}^{2}+a_{o}{}^{2}+2a_{r}a_{o}\cos\left[\varphi_{r}\left(x,y\right)-\varphi_{o}\left(x,y\right)\right]$$

The interference fringes formed by this interference contain both amplitude and phase information of the object light. The amplitude information of the object light wave is obtained from the change in the visibility of the interference fringes, and the phase information of the interference fringes is obtained from the shape and spacing of the interference fringes.

The computed complex field in the CGH plane is then encoded to obtain the complex transmittance function $t\left(x,y\right)$ of the CGH:

(4) $$t\left(x,y\right)=k_{0}+k_{1}I\left(x,y\right)$$

Finally, the complex transmittance function is realised onto a specific optical element, a process that uses computer-controlled electron beam lithography equipment to replicate the pattern onto a CGH substrate, which, in turn, produces a diffractive optical element with $t\left(x,y\right)$ as the diffraction pattern.

The above process describes the principle of preparation of standard wavefront calculations, wavefront storage medium CGH diffractive optical lenses based on the results of the optical design. Since the design process of the hologram is done on a computer, it is possible to obtain any diffraction pattern of the optical mirror that can be described mathematically. Therefore, the CGH technique is highly flexible in generating freeform wavefronts.

#### 4.1.2. Wavefront Reproduction

Once the hologram has been recorded and stored, the second step involves the process of wavefront reconstruction. The CGH diffractive optical element, obtained through computation and fabrication during the first step, can be considered as a transmissive screen with a transmission coefficient of t, acting as a composite grating. This hologram records the interference patterns formed by the coherent superposition of the ideal object wave and the reference wave in a computer-generated manner. When the hologram is illuminated with the reference wave under the same conditions, the diffracted light field behind the hologram contains the reconstructed object wave, which corresponds to the standard wavefront of the object under investigation.

**Figure 7:**
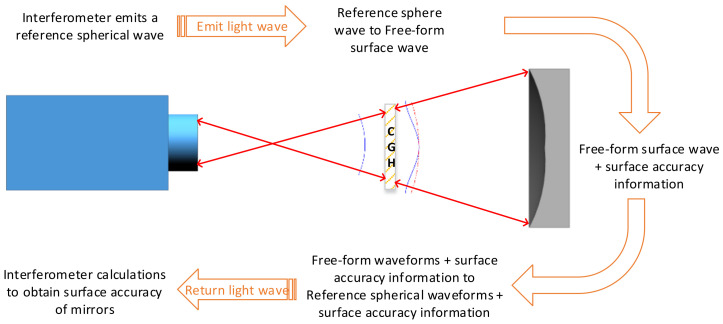
Optical circuit diagram of face shape accuracy detection with CGH as a compensator.

By utilising computer-generated holography (CGH), it is possible to recreate the ability of the inspected optical mirror to reconstruct standard wavefronts, allowing CGH to be used as a compensator for the assessment of the freeform reflective mirrors. The schematic diagram of the inspection optical path is shown in Figure 7.

The detection process involves the utilisation of an interferometer to emit a reference light wave. This reference light wave, after passing through the computer-generated hologram (CGH), undergoes diffraction to achieve the transformation from the reference light wave to the ideal wavefront of the freeform surface. Subsequently, the light wave reaches the surface of the mirror under testing, capturing the mirror’s surface wavefront information upon its return. The returned freeform wavefront is then converted back to a reference light wave carrying the mirror’s wavefront information. Finally, through the measurements and calculations performed by the interferometer, the surface profile information of the mirror under testing is obtained.

**Figure 8:**
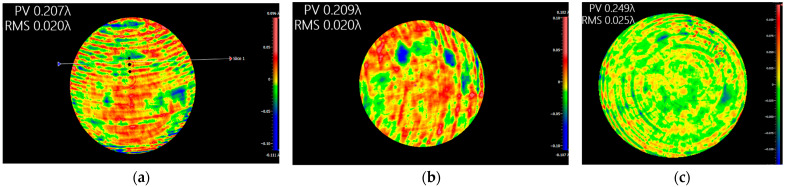
The surface shape detection results of three freeform mirrors in the system, where (**a**–**c**) are the surface shape accuracy detection results of the primary, secondary, and tertiary mirrors, respectively.

It is worth noting that the parameters of the reference light wave emitted by the interferometer should be consistent with those of the reference light wave used during the CGH design. In our study, we adopted a commercially available interferometer using a light source with a wavelength of 632.8 nm, thus eliminating the restrictions imposed by the interferometer’s light source. This method was successfully employed to fabricate and inspect three freeform surface mirrors in our system. The final reflector surface shape error detection results are shown in Figure 8.

### 4.2. Mounting Method of the Double CGH Common Reference for the Primary and Secondary Mirrors

The previous subsection focused on the detection principle of CGH and the construction of the detection optical path of the freeform mirrors, as well as the detection results. After the completion of the surface shape detection of the monolithic mirrors, the system mounting stage can be entered, and the mounting method of the freeform off-axis triple-reflector first needs to be formulated. The basic idea of off-axis triple-reflector optical system tuning is to use a certain positioning method to adjust the two mirrors in the system to the theoretical position and then adjust the third mirror and the folding mirror, until the system’s wavefront aberration reaches the requirements of the design index of the optical system.

**Figure 9:**
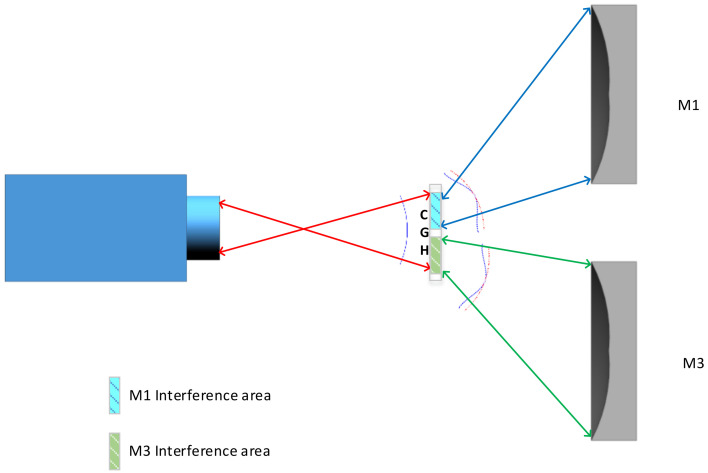
Conventional TMA off-axis SLR system main mirror, three mirrors CGH common reference of the mounting, and adjustment of the optical circuit diagram.

Positioning the first two mirrors into the theoretical position is a key step in the commissioning of an off-axis triple-reflector system. There are two commonly used methods: the first is to use the null lens compensators of the two mirrors for face shape detection to ensure the accuracy of the mirror position by ensuring the accurate positions of the two compensators. Since the null lens of these two mirrors are relatively independent, their positional relationship can only be determined by mechanical positioning, so the implementation of this method is difficult. The second method is the CGH coreference detection method, which, as described in the previous section, can generate wavefronts of almost any shape with precise relative positions between multiple wavefronts, providing a high degree of design flexibility. Therefore, the CGH region for detecting the first mirror and the CGH region for detecting the second mirror can be etched onto the same glass substrate, thereby enabling the detection and positioning of two mirrors by one CGH. Figure 9 shows a schematic of a common off-axis triple-reflector system CGH coreference. Compared with the first method, this method realises the positioning of the two compensators during the CGH fabrication process, so there is no need to introduce a mechanical adjustment link during the commissioning process, which improves the precision of the commissioning. However, it should be noted that the premise of the second method is that the two mirrors to be detected can be surrounded by the light waves emitted by one piece of CGH, so it is not applicable to off-axis triple-reflector systems with complex configurations.

**Figure 10:**
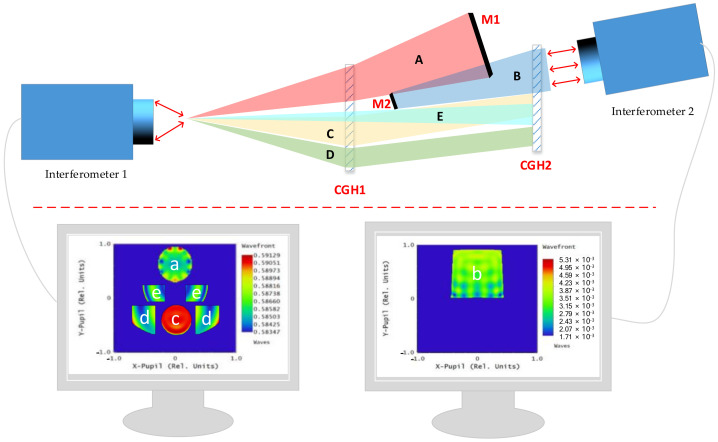
Schematic diagram of the double CGH common reference tuning scheme.

Combined with the structural form of the optical system in Figure 1b, it can be judged that the system is an anisotropically folded spherical structure. Due to the specificity of the layout form, it is difficult to realise the mounting of two mirrors by only one piece of computational holographic grating (CGH). In order to solve this difficulty, we propose a dual CGH coreference mounting scheme, the specific layout of which is shown in Figure 10.

The main idea of the mounting scheme for this off-axis triple-reflector system is to adjust the primary and secondary mirrors in the system to their theoretical positions before proceeding with the mounting of the triple and folding mirrors. As mentioned before, due to the special configuration of the system, it is difficult to use one piece of CGH to locate the two mirrors. Therefore, we adopt an innovative dual CGH coreference mounting method. The specific scheme is shown in Figure 10. Firstly, interferometer 1, CGH1, and CGH2 are used to form the alignment optical path of the dual CGH. Among them, optical path C, optical path D, and optical path E are used for rotational alignment, tilt alignment, and displacement alignment of the relative positions between the two CGHs, respectively. By continuously fine-tuning the relative positions between CGH1 and CGH2, interference fringes c, d, and e are eventually converged to the zero-level fringes, thereby determining the theoretical positions of CGH1 and CGH2. In addition to the inscribed regions with alignment functions, the two CGHs also contain detection inscribed regions for the primary and secondary mirrors. Therefore, after the completion of the precise alignment, the two CGHs can also be used for detecting the positional alignment of the primary and secondary mirrors. Then, interferometer 1, CGH1, and the primary mirror are utilised to form detection optical path A of the primary mirror surface shape, and the primary mirror surface shape is adjusted to the optimum, i.e., the theoretical position of the primary mirror is found by fine adjustment of the primary mirror. Similarly, interferometer 2, CGH2, and the secondary mirror are used to form detection optical path B of the secondary mirror face shape, and the secondary mirror face shape is adjusted to the optimum by fine adjustment of the secondary mirror, i.e., the theoretical position of the secondary mirror is found. Through the above alignment process, the theoretical positional relationship between the primary mirror and the secondary mirror can be determined.

The dual CGH common reference alignment method offers improved design flexibility compared to the single CGH alignment method. However, it introduces a step of aligning two CGHs, which introduces a certain amount of misalignment. To further demonstrate the feasibility of the dual CGH common reference alignment method, we comprehensively considered and analysed the errors generated during the CGH fabrication process and system alignment process. Table 7 presents the accumulated misalignment introduced by each step and the allowable misalignment of the primary and secondary mirrors in the optical system design. From Table 7, it can be observed that the errors generated by each step, when accumulated, still remain below the allowable tolerances specified in the optical design. Therefore, we can conclude that the dual CGH common reference alignment method meets the application requirements of this optical system.

The proposed double CGH coreference mounting method is not only instructive for the mounting of optical systems with spherical configurations but also provides useful ideas for more complex optical systems based on its design flexibility.

### 4.3. Experimental Verification

#### 4.3.1. Primary and Secondary Mirror Positioning and Mounting Test

**Figure 11:**
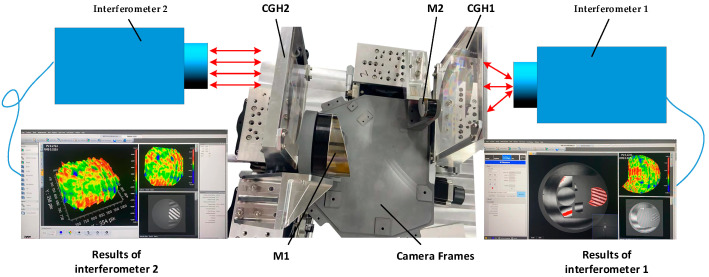
Primary and secondary mirror positioning inspection chart.

According to the development of the mounting method, we further carry out the implementation of the mounting programme: firstly, we carry out the construction of the positioning optical path of the primary and secondary mirrors in the system, which consists of the primary mirror, the secondary mirror, CGH1, CGH2, interferometer 1, and interferometer 2, and the details are shown in Figure 11.

**Figure 12:**
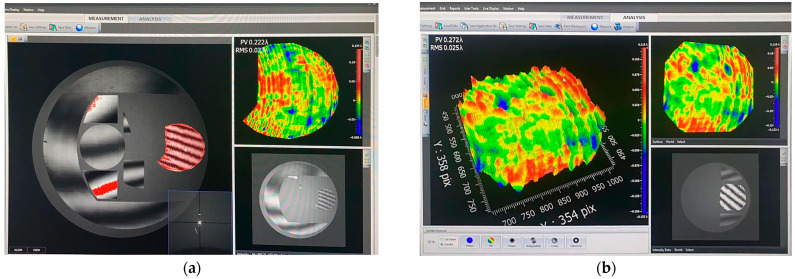
This is the results of two interferometer tests, where (**a**) is the test results of interferometer 1 and (**b**) is the test results of interferometer 2.

The primary and secondary mirror mounting system is demonstrated in Figure 11, and the closed-loop adjustment of the system was achieved by continuously observing the feedback data from the two interferometers. After precision adjustment, the test data of interferometer 1 is shown in Figure 12a, which shows that the interference fringes in all alignment regions are adjusted to the zero-level fringes, which indicates that CGH1, CGH2 and interferometer 1 have reached the theoretical position. The interferometer calculation results in the detection region of the primary mirror show that the face shape accuracy is 0.023 wavelengths, which indicates that the primary mirror has also been adjusted to the theoretical position. Meanwhile, the test data of interferometer 2 is shown in Figure 12b, which indicates that CGH2 and interferometer 2 have reached the theoretical position with the secondary mirror face shape accuracy of 0.025 wavelength. The test results of the two interferometers are very close to the design results, which means that the relative positions of the primary and secondary mirror have reached the theoretical requirements of the optical system design. Next, the camera frame can be adjusted to fix the primary and secondary mirrors to the camera frame.

#### 4.3.2. System Integration and Testing

**Figure 13:**
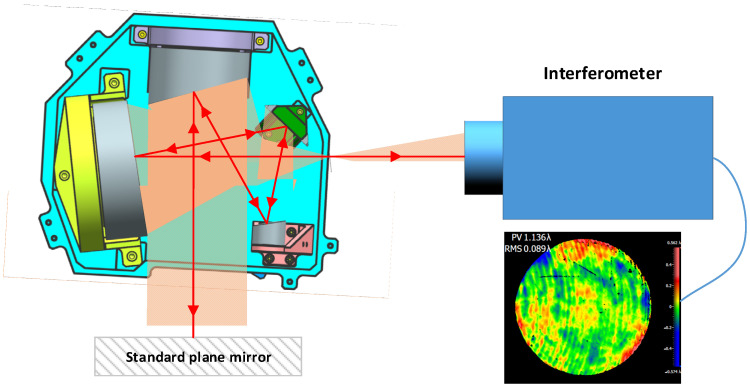
Optical path diagram for the system mounting.

After completing the mounting of the primary and secondary mirrors in the off-axis triple mirror system, the next step is to put the triple mirror and folding mirror into the system for systematic mounting. We build the optical path for the system mounting, as shown in Figure 13: first, we send out a standard spherical wave through the interferometer, and then, the light wave passes through the optical system and returns through the standard plane mirror and is detected by the interferometer to obtain the wavefront difference data of the system. We use the wavefront aberration as the feedback data and keep adjusting the three mirrors and folding mirrors in the system until the wavefront aberration data reach the design requirements of the optical system. Through precise alignment, we finally make the system wavefront aberration reach 1/11 wavelengths and complete the mounting of the optical system. Our test results are shown below. Through the final test results of the system mounting, we verified the feasibility of the dual CGH coreference mounting scheme.

**Figure 14:**
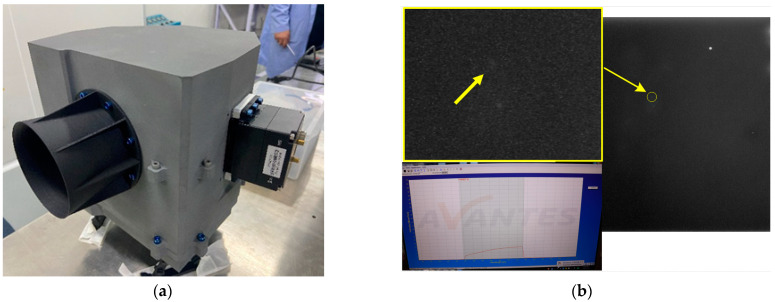
This is the picture and imaging test results of the space camera after completing the integration of the whole camera, where (**a**) is the photo of the whole camera, and (**b**) is the results of the magnitude test.

After the mounting of the optics is complete, the focal plane assembly is integrated into the optics to finalise the integration of the whole machine. The integrated whole camera is shown in Figure 14a.

In order to verify the capability of the payload in space target detection, we conducted a test of the magnitude detection capability using a standard star simulator. By using the star map simulator, we generated a star map containing the standard 14th magnitude and transmitted it to the payload through a parallel light pipe to simulate the detection of a long-range space target. By testing, we were able to judge its magnitude detection capability. The test results are shown in Figure 14b, indicating that the camera can identify space targets of the 14th magnitude and meet the requirements of the system specifications. This further verifies the results of the optic-mechanical system optimisation design and the reasonable feasibility of the optical system mounting scheme.

## 5. Discussion

In this study, the design optimisation of the optic-mechanical system for space optical loads and the integration of the whole machine were carried out, starting from an ultra-compact fully freeform spherical heterostructure optical system. In terms of the optomechanical design, the optomechanical integration optimisation design method was adopted, focusing on solving the difficulties of the ultra-lightweight requirement of the space optical load and the stable support of the optical system. After the optimised design, the weight of the whole system is was 10 kg, and the morphology of a single reflector mirror met the requirement of 1/40 λ under various loading conditions, such as gravitational release into orbit and temperature changes. In terms of system mounting, the difficulty in the mounting and positioning of the spherical heterogeneous optical system was explored, and a double CGH common reference mounting method was proposed. This method achieved accurate positioning of the special configuration optical system, and the system wavefront difference was better than 1/10 λ, which met the requirements of the optical system. After completing the focal plane integration, the imaging capability of the camera was tested to further verify the feasibility of the optic-mechanical system design and mounting scheme. The optomechanical integration optimisation method proposed in this paper is of significance as a guide for achieving the stabilisation support technology of the optical system for ultra-lightweight and ultra-compact space optical loads. Meanwhile, the optical mounting scheme proposed in this study also has good design flexibility and provides a new mounting idea for the engineering realisation of multi-reflector systems with complex special configurations.

## 6. Conclusions

This article proposed an optimised design and optical alignment method for a fully off-axis three-mirror freeform optical system, enabling the realisation of an ultra-lightweight and ultra-compact space camera. The optical-mechanical integrated analysis method was employed to achieve the optimised design of the optical-mechanical system, resulting in a total weight of less than 10 kg. Furthermore, a novel dual CGH common-based alignment method was introduced, which was validated through alignment and testing experiments, demonstrating a system wavefront error better than 1/10 λ. The final star rating tests met the requirements of the optical system design. These research findings provide new technical solutions for the development of ultra-compact space cameras, with significant implications for downsizing space surveillance systems.

## Figures and Tables

**Table 1:** Field of view parameters of the current mainstream space-based surveillance cameras.

Nations	Surveillance Camera	Field of View
USA	SBIRS	0.44° × 0.44° (gaze-based) 10° × 20° (scanning type)
USA	MSX satellite infrared camera	1° × 3° (scanning type)
USA	MSX satellite visible camera	1.4° × 6.6° (gaze-based)
USA	SBSS satellite optical payload	1.4° × 6.6° (gaze-based)
USA	STARE satellite optical payload	2.08° × 1.67° (gaze-based)
Canada	Sapphire satellite payload	1.4° (gaze-based)
Canada	NEOSSat Programme	0.85° × 0.85° (gaze-based)
German	Asteroid Finder payload	2° × 2° (gaze-based)

**Table 2:** Parameters of the off-axis triple-reflector full freeform optical system.

Parameter Name	Date
Entrance pupil diameter	66.67 mm
System Focus	200 mm
Field of view	4° × 4°
limit magnitude	14th magnitude

**Table 3:** Properties of materials commonly used in space optics mirrors.

Type of Material	Density (10^−6^ kg/mm^3^)	CTE (10^−6^/°C)	Thermal Conductivity (W/(mm·°C))	Elastic Modulus (MPa)	Processability
SiC	3.05	2.5	0.185	392,000	err
Aluminium	2.8	21.4	0.121	66,640	superior
Microcrystalline Glass	2.53	0.1	0.00164	90,300	favourable
Quartz Glasses	2.2	0.55	0.00138	730,098.2	favourable
Beryllium aluminium alloy	2.1	13.9	0.212	1,930,000	superior

**Table 4:** Design variables and optimisation results.

Name	a1	a2	a3	b1	b2	b3
Ranges	[2.5–10]	[2–8]	[3–10]	[3.5–12]	[3.5–10]	[3.5–12]
Initialization	3 mm	5 mm	5 mm	6 mm	5 mm	10 mm
Optimization	4.8 mm	4.1 mm	5.8 mm	4.9 mm	3.7 mm	6.7 mm
Rounding value	5 mm	4 mm	6 mm	5 mm	4 mm	7 mm
Shape accuracy	0.018 λ (Xg), 0.017 λ (Yg), 0.019 λ (Zg), 0.019 λ (5 °C)					
Mass	0.563 kg					

**Table 5:** Optimisation results of the mirror shape accuracy for each operating condition.

Mirrors	Xg	Yg	Zg	5 °C
M1	0.018 λ	0.017 λ	0.019 λ	0.019 λ
M2	0.016 λ	0.015 λ	0.017 λ	0.016 λ
M3	0.019 λ	0.018 λ	0.018 λ	0.019 λ
M4	0.014 λ	0.012 λ	0.016 λ	0.017 λ

**Table 6:** Final integration analysis results for the optic-mechanical system.

Mirrors	Design Indicators	Xg	Yg	Zg	5 °C
Wavefront difference of the system	0.1 λ	0.102 λ	0.104 λ	0.108 λ	0.11 λ
MTF	0.6	0.57	0.57	0.59	0.58
Mass/Size		9.61 kg/222 mm × 290 mm × 279 mm			

**Table 7:** Double CGH manufacturing and mounting error analysis.

Name	Error Category	Error Value	Misalignment between M1 and M2	Error Synthesis	Tolerance of Optical System
Positional accuracy of interferometer 1 with CGH1	Position accuracy X/Y	<1 μm	<1 μm	<9 μm	<11 μm
	Position accuracy Z	<5 μm	<5 μm		
Positional accuracy of interferometer 2 with CGH2	Position accuracy X/Y	<1 μm	<1 μm		
	Position accuracy Z	<5 μm	<5 μm		
Positional accuracy of CGH1 with CGH2	Position accuracy X/Y	<1 μm	<1 μm		
	Position accuracy Z	<5 μm	<5 μm		
Angular accuracy of CGH1 with CGH2	Rotation accuracy X/Y	<1″	<1″	<2″	<5″

## Data Availability

The data that support the findings of this study are included and are available from the corresponding author.
